# Molecular Mechanisms of P2X Receptor Desensitization

**DOI:** 10.1063/4.0000961

**Published:** 2025-10-27

**Authors:** Steven E. Mansoor

**Affiliations:** Oregon Health & Science University, Portland, OR, USA

## Abstract

Extracellular ATP serves as a crucial signaling molecule, present in varying concentrations across diverse cellular environments. The P2X receptor (P2XR) family, which recognizes extracellular ATP, consists of seven subtypes (P2X1R - P2X7R) that form functional homo- and hetero- trimeric ion channels. These receptors are activated by distinct concentrations of extracellular ATP, ranging from low nanomolar to high micromolar levels, and are expressed in numerous cell types. They play key roles in a variety of pathophysiological conditions affecting the central nervous, immune, and cardiovascular systems. Beyond differences in ATP sensitivity, the kinetics of ion channel gating in response to agonists vary significantly among P2XR subtypes. For example, P2X1Rs and P2X3Rs exhibit rapid desensitization (milliseconds), while P2X2Rs and P2X4Rs undergo slower desensitization (seconds), and P2X7Rs show little to no desensitization. Although membrane-proximal regions within the cytoplasmic termini have long been known to influence P2XR desensitization, the lack of structural data for these domains in any P2XR subtype hindered the development of a detailed molecular explanation for the diverse desensitization profiles. Furthermore, the lack of desensitization observed in P2X7R remained particularly puzzling. In 2016, the first crystal structures of P2X3R in three functional conformational states, including a desensitized state, revealed a novel desensitization mechanism, termed the “helical recoil” model. Later, in 2019, Cryo-EM structures of full-length P2X7R not only confirmed this model but also provided additional insight into the mechanisms that prevent desensitization in P2X7Rs. Recent Cryo-EM structures of the human P2X4R not only refine the helical recoil model but also identify a putative lipid-binding site in the cytoplasmic domain that further modulates desensitization kinetics. Over the past decade, structural biology has significantly advanced our understanding of P2XR structure and function, particularly in relation to the molecular mechanisms of receptor gating.

